# Pregnancies among women living with HIV using contraceptives and antiretroviral therapy in western Kenya: a retrospective, cohort study

**DOI:** 10.1186/s12916-021-02043-z

**Published:** 2021-08-13

**Authors:** Rena C. Patel, Gustavo Amorim, Beatrice Jakait, Bryan E. Shepherd, A. Rain Mocello, Beverly Musick, Caitlin Bernard, Maricianah Onono, Elizabeth A. Bukusi, Kara Wools-Kaloustian, Craig R. Cohen, Constantin T. Yiannoutsos, on behalf of the Implant/Efavirenz Study Group and the East Africa IeDEA Regional Consortium

**Affiliations:** 1 Division of Allergy and Infectious Diseases, Department of Medicine and Department of Global Health, University of Washington, UW Box 359927, 325 Ninth Avenue, Seattle, WA 98104 USA; 2 Department of Biostatistics, Vanderbilt University, Nashville, TN USA; 3 Moi Teaching & Referral Hospital/Moi University & Academic Model Providing Access to Healthcare (AMPATH), Eldoret, Kenya; 4 Bixby Center for Global Reproductive Health and Department of Obstetrics, Gynecology & Reproductive Health, University of California, San Francisco, San Francisco, CA USA; 5 Department of Biostatistics, School of Medicine, Indiana University, Indianapolis, IN USA; 6 Division of Family Planning, Department of Obstetrics & Gynecology, Indiana University School of Medicine, Indianapolis, IN USA; 7 Centre for Microbiology Research, Kenya Medical Research Institute, Nairobi, Kenya; 8 Department of Medicine, Indiana University, Indianapolis, IN USA; 9 Department of Biostatistics, R.M. Fairbanks School of Public Health, Indiana University, Indianapolis, IN USA

**Keywords:** HIV, Women living with HIV, Contraception, Efavirenz, Implant, Pregnancy

## Abstract

**Background:**

Preventing unintended pregnancies is paramount for women living with HIV (WLHIV). Previous studies have suggested that efavirenz-containing antiretroviral therapy (ART) reduces contraceptive effectiveness of implants, but there are uncertainties regarding the quality of the electronic medical record (EMR) data used in these prior studies.

**Methods:**

We conducted a retrospective, cohort study of EMR data from 2011 to 2015 among WLHIV of reproductive age accessing HIV care in public facilities in western Kenya. We validated a large subsample of records with manual chart review and telephone interviews. We estimated adjusted incidence rate ratios (aIRRs) with Poisson regression accounting for the validation sampling using inverse probability weighting and generalized raking.

**Results:**

A total of 85,324 women contributed a total of 170,845 women-years (w-y) of observation time; a subset of 5080 women had their charts reviewed, and 1285 underwent interviews. Among implant users, the aIRR of pregnancy for efavirenz- vs. nevirapine-containing ART was 1.9 (95% CI 1.6, 2.4) using EMR data only and 3.2 (95% CI 1.8, 5.7) when additionally using both chart review and interview validated data. Among efavirenz users, the aIRR of pregnancy for depomedroxyprogesterone acetate (DMPA) vs. implant use was 1.8 (95% CI 1.5, 2.1) in EMR only and 2.4 (95% CI 1.0, 6.1) using validated data.

**Conclusion:**

Pregnancy rates are higher when contraceptive implants are concomitantly used with efavirenz-containing ART, though rates were similar to leading alternative contraceptive methods such as DMPA. Our data provides policymakers, program staff, and WLHIV greater confidence in guiding their decision-making around contraceptive and ART options. Our novel, 3-phase validation sampling provides an innovative tool for using routine EMR data to improve the robustness of data quality.

**Supplementary Information:**

The online version contains supplementary material available at 10.1186/s12916-021-02043-z.

## Introduction

Women account for greater than half of the estimated 36 million people living with HIV and two million new HIV infections annually worldwide [1]. For the vast majority of women living with HIV (WLHIV), preventing unintended pregnancies is not only paramount for the woman’s health but also important for the prevention of mother-to-child transmission of HIV. Great strides have been made in improving universal access to antiretroviral therapy (ART) and the provision of effective contraception in resource-limited settings [2, 3]. For example, the use of subdermal contraceptive implants, which are the most effective contraceptive method with failure rates < 1%, has risen from a prevalence of 1.7% in 2003 to 18.1% in 2016 among married women in Kenya [4]. Yet, limited options for ART use and effective contraceptive methods exist in these settings. The recent scare with dolutegravir exposure periconception and possible increased risk of neural tube defects [5] underscores the limited options for ART and the difficult family planning decision-making process WLHIV, their providers, and policymakers face in resource-limited settings.

In light of the possible association between dolutegravir and birth defects, many national HIV treatment programs initially defaulted to recommending efavirenz-containing ART as the best option for women of reproductive potential [6]. However, our prior work, a retrospective analysis of electronic medical record (EMR) data from one program in western Kenya, has demonstrated reduced effectiveness of contraceptive implants when concurrently used with efavirenz-containing ART [7]. An increasing number of observational and pharmacokinetic studies have supported these findings [8–12], indicating that the likely cause is drug-drug interactions with efavirenz increasing the metabolism of the exogenous progestin in the contraceptive implants leading to reduced concentrations of the circulating progestin.

Although the EMR is a great source of data for questions regarding a large number of patients or rare outcomes, and surveillance of adverse pregnancy or neonatal outcomes are likely best ascertained using routine data systems, there are recognized challenges to using routinely collected EMR data for research purposes, particularly due to data quality concerns [13, 14]. Some studies, including several in the HIV/AIDS literature, have seen dramatic changes in estimates after data validation [15–19]. Nonetheless, significant investments are being, and will continue to be, made in health informatics systems, including in resource-limited settings; for example, Kenya now supports a robust national EMR system [20, 21]. It is critical to know whether the initial findings suggesting a reduced effectiveness of contraceptive implants with concomitant efavirenz-containing ART can be substantiated with higher quality data. Thus, to better estimate the associations between contraceptive use, ART regimens, and pregnancy, we conducted a follow-up study expanding our study sample to include another large HIV treatment program in western Kenya as well as incorporating a three-phase validation study, reviewing over 5000 charts and conducting over 1000 phone interviews to establish accuracy of the EMR in our primary exposures and outcome of interest.

## Methods

### Study setting, site, and population

We conducted a retrospective analysis of a longitudinal cohort of WLHIV from 15 to 45 years of age followed from January 1, 2011, to December 31, 2015, at two HIV treatment programs in western Kenya affiliated with the East Africa International Epidemiology Databases to Evaluate AIDS (EA-IeDEA). These two President’s Emergency Plan for AIDS Relief (PEPFAR)-sponsored HIV treatment programs, Academic Model Providing Access to Healthcare (AMPATH) and Family AIDS Care & Education Services (FACES), supported care for approximately 72,000 and 50,000 individuals living with HIV in western Kenya, respectively, during the study period. The chart review and telephone interviews were conducted from April 2016 to March 2017.

The Human Subjects Division at the University of Washington; Indiana University Institutional Review Board; Committee on Human Research at the University of California, San Francisco; Institutional Research and Ethics Committee at Moi University/Moi Teaching and Referral Hospital, Ethical Review Committee at Kenya Medical Research Institute; and US Centers for Disease Control and Prevention approved this research.

### Observation periods and censoring

An observation period that began at first clinical visit captured in the EMR for a woman on or after January 1, 2011, would change when a contraceptive method or ART regimen or both changed or the woman became pregnant, and the final observation period would end at the last visit on or before December 31, 2015. Thus, each observation could span multiple clinical visits, and women with only one visit in the EMR would not contribute any person-time to our study. We made no efforts to track women who were potentially lost to follow-up, transferred their care out to another facility, or died. Additional details can be found elsewhere [22].

In the AMPATH dataset, women were considered not to be at risk for a subsequent pregnancy, hence, censored, for the duration of the current pregnancy as indicated by the pregnancy outcome records (miscarriage, abortion, or preterm or term delivery). In the FACES dataset, however, such information was unavailable, and therefore, women who became pregnant were considered not to be at risk for a subsequent pregnancy for 38 weeks, starting from the date of likely conception, i.e., the period of duration for a full-term birth. After the pregnancy, the women were considered to be at risk again and could contribute multiple pregnancies to our dataset.

### Variable definitions

#### Exposures

Contraceptive method was documented at each clinic visit and then categorized as follows: (1) implants, which may have included information on specific types of etonogestrel-containing (e.g., Implanon®/Implanon-NXT®/Nexplanon®) or levonorgestrel-containing (e.g., Jadelle®) implants; (2) depomedroxyprogesterone acetate (DMPA); (3) oral contraceptive pills (OCPs), including combined oral contraceptive or progestin-only pills; (4) other more effective contraceptive (MEC) methods such as intrauterine devices (IUDs) and permanent methods; (5) less effective contraceptive (LEC) methods, such as male and female condoms and “natural” contraceptive methods (withdrawal and rhythm); or (6) no contraceptive method. When multiple methods were documented at the same visit, the contraceptive method was assigned according to the following hierarchy: MEC over implants over DMPA over OCPs over LEC.

During the start of the study period in Kenya, nevirapine- or efavirenz-containing ART were the recommended first-line ART, but by early 2013, efavirenz-containing ART became the recommended first-line ART [23] and universal ART was not recommended until 2016 [24]. The ART regimen was documented at each visit and was categorized as follows: (1) efavirenz-containing ART; (2) nevirapine-containing ART; (3) protease inhibitor (PI)-containing ART; (4) nucleos(t)ide reverse transcriptase inhibitors (NRTIs) only-containing ART; (5) a combination ART regimen containing two or more of efavirenz, nevirapine, or PIs; or (6) no ART. We defined an “ART regimen” as at least a three-drug combination of antiretrovirals. Due to few person-years in ART regimen categories 3 through 5, observations in these categories were dropped before conducting this analysis. We chose the use of nevirapine-containing ART as the reference category for ART comparisons across contraceptive methods, as the alternative option of no ART is not clinically meaningful in the era of universal ART use.

#### Outcome

Our primary outcome was incident pregnancy documented in medical records by a clinical diagnosis, through self-reports or presenting while gravid. Neither urine nor serum tests were routinely used to confirm clinically suspected pregnancies or prior to implant placement in the study setting. We estimated the date of incident pregnancy as the date of likely conception based on reports of last menstrual period, estimated gestational age, or estimated date of delivery. We assumed the contraceptive method was still being used if the method is a permanent method, not noted to be explicitly removed (in the case of implants or IUDs), or another contraceptive method has not been initiated prior to the pregnancy (applicable to all methods). In order to identify pregnancies that may have been conceived towards the end of our study period but not yet clinically detected, we tracked reported pregnancies for another nine months past December 2015. Additional details can be found in supplementary text A.

#### Covariates

A priori we included age, marital status, number of living children under 14 years of age (as a proxy for parity since these data were not directly available), education level, CD4 cell count, WHO clinical stage of HIV disease, body mass index (BMI), use of anti-tuberculosis (TB) medications, calendar time, and program as adjusting variables. Additional details on covariates can be found in supplementary text A.

### Data validation via three-phase sampling

We designed a 3-phase sampling scheme for data validation, to overcome potential limitations in data collection and entry errors in the EMR (supplementary figure 1), adapted from 2-phase sampling schemes [25, 26]. The first phase sample consisted of routine data collected from the EMR. Our second phase sample consisted of a manual chart review for a subsample of patient records from the EMR. We randomly selected records for chart review by categorizing records into 32 categories based on combinations of contraceptive method (implant, DMPA, MEC, none), ART exposure (efavirenz, nevirapine, PI, no ART), and pregnancy status (pregnancy, no pregnancy). We over-sampled records from certain categories of particular interest. Charts were reviewed by trained research assistants. Our third phase sample consisted of telephone interviews for a non-random subset of women for whom we completed chart reviews. Priority was given to interviewing women noted to become pregnant while using an implant, women not pregnant while using an implant, women pregnant while using DMPA, and women not pregnant while using DMPA, in that order. Phone interviews were performed by research assistants with a standardized telephone script and after obtaining verbal consent. We removed observation time in the chart review and telephone interview data before and/or after observation time in the EMR for the weighted analyses. Our goal was to validate only the primary exposures (contraceptive method and ART regimen) and outcome (incident pregnancy) of interest in this study, so we ascertained only data pertaining to these three variables in the second and third sampling phases. When differing values were generated for these three variables in the three datasets, we generally gave priority to the values recorded in the telephone interview dataset for use in analyses. However, for the ART regimen, when the telephone interview dataset indicated that the woman could not recall or was unsure of her ART regimen, we used the ART regimen values observed in the chart review dataset. Details for each individual phase and its methods are found in supplementary text A, figures 2 and 3, and table 1.

### Statistical analysis

We present frequencies and proportions for categorical variables and median and interquartile range (IQR) for continuous variables. We imputed missing data in the EMR, which ranged in freqeuncy from 0.4 to 25.7% (Table 1), using multiple imputations by iterative chained equations, with all model covariates, contraceptive method, ART regimen, and pregnancy as predictors and, for time-varying variables, including the next and preceding non-missing values. We calculated adjusted incident rate ratios (aIRRs) using Poisson models with interaction terms between contraceptive method and ART categories, program, the various covariates mentioned above, and robust standard errors. All estimates are presented with interaction terms since the interaction terms are central to the study question.

**Table 1:** General characteristics of women sampled in each phase, based on woman-years contributed to each sample phase (proportion (%) or median (IQR))

Characteristics^a^	EMR (1st phase)	Chart review (2nd phase)	Telephone interview (3rd phase)
Total women-years, total number of women	170,844.6, 85,324	16,989.8, 4971	5636.9, 1243
Number of observations per woman (median (IQR))	4.0 (2.0–7.0)	3.0 (2.0–4.0)	4.0 (3.0–5.0)
Total observation time per woman in years (median (IQR))	2.0 (0.5–3.4)	3.9 (2.5–4.6)	4.6 (4.2–5.0)
Age at the start of the observation period (median (IQR))	33.3 (28.0–38.5)	31.3 (27.1–35.9)	30.8 (27.1–35.1)
Contraceptive method			
Implant	11,978.6 (7.0)	6694.6 (39.4)	2151.7 (38.2)
DMPA	22,749.3 (13.3)	4228.4 (24.9)	988.7 (17.5)
OCP	2252.7 (1.3)	333.7 (2.0)	88.3 (1.6)
MEC	6814.4 (4.0)	790.6 (4.7)	157.3 (2.8)
LEC	41,237.0 (24.1)	1643.5 (9.7)	303.9 (5.4)
No method	82,939.8 (48.6)	3299.0 (19.4)	1940.6 (34.4)
Missing	2872.8 (1.7)	0.0 (0.0)	6.4 (0.1)
ART regimen			
Nevirapine-containing	83,435.5 (48.8)	7889.4 (46.4)	2068.4 (36.7)
Efavirenz-containing	42,170.2 (24.7)	5015.0 (29.5)	1352.2 (24.0)
PI-containing	10,044.0 (5.9)	1206.4 (7.1)	355.5 (6.3)
No ART	31,928.3 (18.7)	2843.7 (16.7)	1671.7 (29.7)
Others	202.8 (0.1)	30.6 (0.2)	14.3 (0.3)
ART unknown	0.0 (0.0)	4.7 (0.0)	173.3 (3.1)
Missing	3063.8 (1.8)	0.0 (0.0)	1.5 (0.0)
Education level			
Completed college	1228.4 (0.7)	44.9 (0.3)	9.2 (0.2)
Some college/university	5482.1 (3.2)	570.5 (3.4)	255.8 (4.5)
Completed secondary	16,106.7 (9.4)	1164.3 (6.9)	398.1 (7.1)
Some secondary	19,938.6 (11.7)	1928.2 (11.4)	636.1 (11.3)
Completed primary	23,696.3 (13.9)	2312.3 (13.6)	595.2 (10.6)
Some primary	59,825.4 (35.0)	6352.7 (37.4)	1555.6 (27.6)
None	696.1 (0.4)	14.7 (0.1)	5.0 (0.1)
Missing	43,871.1 (25.7)	4602.2 (27.1)	2182.0 (38.7)
Marital status			
Not married but living with a partner	1612.2 (0.9)	184.1 (1.1)	62.1 (1.1)
Never married and not living with a partner	18,649.0 (10.9)	1003.5 (5.9)	301.2 (5.3)
Separated/divorced	17,239.8 (10.1)	1636.8 (9.6)	705.4 (12.5)
Legally married	82,097.1 (48.1)	9950.0 (58.6)	3097.7 (55.0)
Widowed	28,082.3 (16.4)	1786.3 (10.5)	450.2 (8.0)
Missing	23,164.2 (13.6)	2429.1 (14.3)	1020.3 (18.1)
Number of living children			
0	1598.6 (9.4)	23,360.3 (13.7)	1758.6 (31.2)
1+	11,039.0 (65.0)	94,781.3 (55.5)	3878.3 (68.8)
Missing	4352.2 (25.6)	52,703.1 (30.9)	0.0 (0.0)
WHO clinical stage			
1	63,905.1 (37.4)	7649.5 (45.0)	2616.4 (46.4)
2	46,840.8 (27.4)	4696.9 (27.7)	1584.6 (28.1)
3	48,634.6 (28.5)	3834.1 (22.6)	1162.0 (20.6)
4	10,222.6 (6.0)	787.3 (4.6)	264.4 (4.7)
Missing	1241.6 (0.7)	21.9 (0.1)	9.6 (0.2)
CD4 cell count			
Median (IQR)	428.0 (274.0–609.0)	484.0 (339.0–679.0)	494.9 (353.0–678.4)
< 50	2671.8 (1.6)	97.6 (0.6)	61.7 (1.1)
50–199	14,597.8 (8.5)	924.8 (5.4)	296.1 (5.3)
200–349	32,166.7 (18.8)	2740.8 (16.1)	871.6 (15.5)
350–499	41,537.1 (24.3)	4067.5 (23.9)	1378.6 (24.5)
500+	67,998.3 (39.8)	8104.9 (47.7)	2710.9 (48.1)
Missing	11,872.9 (7.0)	1054.2 (6.2)	318.0 (5.6)
Weight (kg)			
Median (IQR)	57.0 (51.1–64.5)	58.2 (52.5–65.0)	59.0 (53.0–66.3)
< 50	30,809.0 (18.0)	2436.3 (14.3)	730.3 (13.0)
50–59	61,135.7 (35.8)	6337.3 (37.3)	2215.3 (39.3)
60–69	47,672.0 (27.9)	5137.0 (30.2)	1646.7 (29.2)
70+	30,588.7 (17.9)	3025.1 (17.8)	1027.8 (18.2)
Missing	639.1 (0.4)	54.0 (0.3)	16.8 (0.3)
BMI (kg/m^2^)			
Median (IQR)	21.7 (19.6–24.3)	22.0 (20.1–24.4)	22.3 (20.1–24.9)
18.5 (underweight)	18,702.9 (11.0)	1384.1 (8.2)	455.1 (8.1)
18.5–25 (normal weight)	100,289.4 (58.7)	10,900.2 (64.2)	3492.3 (62.0)
25–30 (overweight)	27,602.0 (16.2)	2943.9 (17.3)	1048.4 (18.6)
30+ (obese)	8490.5 (5.0)	760.0 (4.5)	313.3 (5.6)
Missing	15,759.9 (9.2)	1001.6 (5.9)	327.9 (5.8)
Active tuberculosis treatment			
None	163,287.5 (95.6)	15,896.3 (93.6)	5307.0 (94.2)
Active	7557.2 (4.4)	1093.4 (6.4)	329.9 (5.9)
Calendar year			
2011	67,811.2 (39.7)	6433.2 (37.9)	2868.4 (50.9)
2012	49,226.4 (28.8)	4687.0 (27.6)	1159.5 (20.6)
2013	32,343.5 (18.9)	3019.0 (17.8)	817.0 (14.5)
2014	164,25.0 (9.6)	2143.6 (12.6)	550.7 (9.8)
2015	5038.58 (3.0)	707.0 (4.2)	241.3 (4.3)

*ART*, antiretroviral therapy; *EMR*, electronic medical record; *DMPA*, depomedroxyprogesterone acetate; *OCPs*, oral contraceptive pills; *MEC*, other more effective contraceptive methods, such as intrauterine devices (IUDs) and permanent methods; *LEC*, less effective contraceptive methods, such as male or female condoms and “natural” contraceptive methods of withdrawal or rhythm; *PI*, protease inhibitor; *BMI*, body mass index

^a^ART regimen and contraceptive method are considered time-varying exposures. All other variables are ascertained at the start of the ART/contraceptive combination category and assumed constant within an ART/contraceptive combination category, but allowed to vary between ART/contraceptive combination categories

We applied generalized raking inverse probability weighting (IPW) to obtain estimates of the aIRRs using the chart review (second phase) and telephone interview (third phase) data that accounted for the over-sampling of women in certain contraceptive-ART-pregnancy categories [27, 28]. Specifically, we first determined the probability of being selected for the manual chart review, denoted as *p*_*1*_, which was empirically estimated based on the observed proportions of women sampled in each of the 32 contraceptive-ART-pregnancy categories described above. Next, given that a woman’s chart was reviewed, we estimated the probability that she was selected for a telephone interview, denoted as *p*_*2*_. This was done using logistic regression models that included as covariates the categories used to define the priority telephone interviewing strategy (described above). For analyses using chart review data only, data from women with chart validation were assigned a weight 1/*p*_*1*_; for analyses using telephone interviews, data from women with both chart validation and phone interviews were assigned a weight 1/*(p*_*1*_*p*_*2*_*).* To improve the efficiency of our estimates, we applied generalized raking techniques [27], which use auxiliary variables recorded on all women in the EMR to fine-tune these inverse probability weights. Specifically, we calibrated our inverse probability weights using the estimated influence function derived from a Poisson model fit to the unvalidated data [29]. Lastly, we then obtained raking estimates of the aIRRs by fitting a Poisson model to the fully validated data using these calibrated weights and the covariates described above. Under assumptions that missing validation data are missing at random (i.e., that selecting a woman for validation depends only on known characteristics) and that models for these probabilities are correctly specified, these generalized raking IPW estimates of the adjusted IRRs are consistent estimates for the full EMR dataset (i.e., accurately approximate what one would get if one validated all records) [30]. Additional details on statistical methods are found in supplementary text A.

Data were prepared using SAS version 9.3 (SAS Institute, Cary, NC, USA), and analyses were conducted using R version 3.6.1 (R Core Team, Vienna, Austria). Analysis code is publicly available at https://github.com/gustavodecastro/ImplantEFV.

## Results

### General characteristics of cohort

In this analysis, 85,324 women (53,711 from AMPATH and 31,613 from FACES) contributed a total of 170,845 women-years (w-y) of observation time with a median of four observations (IQR 2.0 to 7.0) and 2.0 years of total observation time per woman (IQR 0.5 to 3.4; Table 1). Women had a median age of 33.3 (IQR 28.0 to 38.5) years, 38.9% of the time women had completed primary schooling or a higher level of education, 49.0% of the time women were married or co-habiting, 65.0% of the time had at least one living child, and 64.8% of the time women were in WHO clinical stages 1 or 2. These parameters were generally similar among the women in the second and third phase samples.

Women used implants 7.0%, DMPA 13.3%, OCPs 1.3%, MEC 4.0%, LEC 24.1%, and no contraceptive method 48.6% of the total w-y of observation time. WLHIV used nevirapine-containing ART 48.8%, efavirenz-containing ART 24.7%, PI-containing ART 5.9%, and no ART 18.7% of the total w-y of observation time (Table 1). A total of 12,896 incident pregnancies were observed in the EMR among 11,724 women.

### Relative incidence of pregnancy

The aIRR for pregnancy among implant users for efavirenz- vs. nevirapine-containing ART use in the EMR (first phase), chart review (second phase), and telephone interview (third phase) samples were 1.9 (95% CI 1.6, 2.4), 2.2 (95% CI 1.7, 3.0), and 3.2 (95% CI 1.9, 5.4), respectively, in unweighted analyses that ignored the validation sampling strategy (Table 2). Weighted estimates that accounted for the validation sampling strategy were similar: 2.3 (95% CI 1.5, 3.5) with the chart review only and 3.2 (95% CI 1.8, 5.7) including the telephone interview data (Table 2). Adjusted pregnancy incidence by implant type (e.g., etonogestrel, levonorgestrel, or unknown) and ART regimen, for both unweighted and weighted analyses, were largely similar between the types (supplementary table 1).

**Table 2:** Adjusted pregnancy incident rate ratios per 100 women-years (and 95% CI) by contraceptive method and ART category, individually by each sampling phase ignoring validation sampling strategy (unweighted) and accounting for validation sampling strategy (weighted)

Contraceptivemethod and ARTregimen category	Pregnancy	Women-years	EMR, aIRR^a^ (95% CI)	Chart review, aIRR (95% CI)		Telephone interview, aIRR (95% CI)	
				Unweighted^a^	Weighted^b^	Unweighted^a^	Weighted^b^
Implant	466	11,978.6					
Nevirapine	149	5612.7	Ref	Ref	Ref	Ref	Ref
Efavirenz	187	3244.4	**1.9 (1.6, 2.4)**	**2.2 (1.7, 3.0)**	**2.3 (1.5, 3.5)**	**3.2 (1.9, 5.4)**	**3.2 (1.8, 5.7)**
PI	20	759.7	1.0 (0.6, 1.6)	0.9 (0.5, 1.8)	0.6 (0.2, 1.4)	0.7 (0.2, 2.8)	0.9 (0.3, 2.7)
No ART	110	2344.9	**1.3 (1.0, 1.6)**	1.3 (0.9, 2.0)	0.9 (0.5, 1.5)	1.5 (0.8, 2.8)	1.9 (0.9, 3.7)
DMPA	2150	22,749.3					
Nevirapine	940	10,827.1	Ref	Ref	Ref	Ref	Ref
Efavirenz	512	5000.9	1.1 (0.9, 1.2)	1.2 (0.9, 1.5)	1.0 (0.6, 1.8)	1.0 (0.7, 1.6)	1.0 (0.3, 2.9)
PI	142	1468.7	1.1 (0.9, 1.3)	**1.5 (1.1, 2.1)**	1.3 (0.6, 2.7)	1.0 (0.6, 1.8)	0.8 (0.2, 2.9)
No ART	553	5403.0	**0.9 (0.8, 1.0)**	**1.4 (1.1, 1.7)**	0.9 (0.5, 1.4)	1.0 (0.6, 1.5)	0.9 (0.3, 2.4)

Estimates in bold indicate statistically significant findings

*ART*, antiretroviral therapy; *EMR*, electronic medical record; *DMPA*, depomedroxyprogesterone acetate; *PI*, protease inhibitor

^a^Calculated using Poisson models with interaction terms between ART and contraceptive method categories, program, various covariates (average age within the observation period, average age squared, marital status, education status, any number of living children, WHO clinical stage, CD4 cell count, square root of CD4 cell count, log BMI, square root of log BMI, use of any anti-tuberculosis medications, and calendar time), and robust standard errors. Unweighted analyses ignored the validation sampling strategy

^b^Calculated using generalized raking inverse probability weighting and Poisson models with interaction terms between ART and contraceptive method categories, program, various covariates (average age within the observation period, average age squared, marital status, education status, any number of living children, WHO clinical stage, CD4 cell count, square root of CD4 cell count, log BMI, square root of log BMI, use of any anti-tuberculosis medications, and calendar time), and robust standard errors. Weighed analyses accounted for the validation sampling strategy

The aIRR for pregnancy among DMPA users for efavirenz- vs. nevirapine-containing ART use in the EMR, chart review, and telephone interview were 1.1 (95% CI 0.9, 1.2), 1.2 (0.9 to 1.5), and 1.0 (0.7 to 1.6), respectively, in unweighted analyses and 1.1 (0.6 to 1.8) with the chart review only and 1.0 (0.3 to 2.9) including the telephone interview data in weighted analyses accounting for the validation sampling strategy (Table 2).

**Fig. 1:**
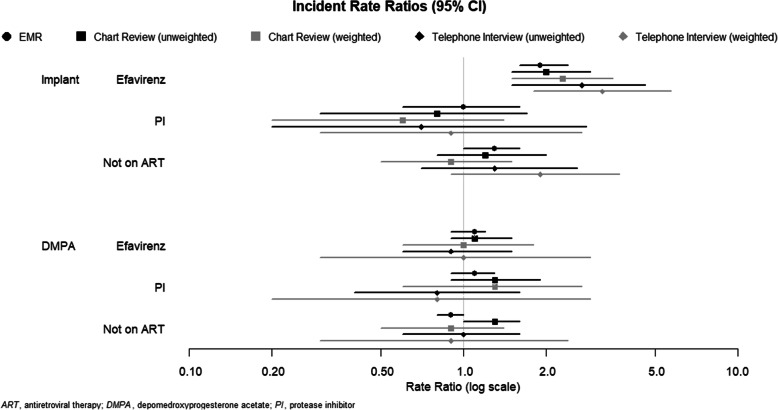
Forest plot of adjusted pregnancy incident rate ratios per 100 women-years by contraceptive method and ART category for each sampling method (reference group is nevirapine-containing ART)

The aIRRs for pregnancy among implant and DMPA users by ART use, both unweighted and weighted, are depicted in Fig. 1.

The aIRR for pregnancy among efavirenz users for DMPA vs. implant use in the EMR, chart review, and telephone interview samples were 1.8 (95% CI 1.5, 2.1), 1.8 (95% CI 1.4, 2.3), and 1.7 (95% CI 1.1, 2.6), respectively, in unweighted analyses and 1.5 (95% CI 0.9, 2.4) for the chart review only and 2.4 (95% CI 1.0, 6.1) including the telephone interview data in weighted analyses (supplementary table 3).

The overall pregnancy and incidence by either contraceptive method or ART regimen or both in the three samples are found in supplementary text B and table 4.

## Discussion

Our study based on EMR data from over 85,000 women, manual chart reviews of over 5000 records, and telephone interviews with over 1000 women confirms prior findings of 2–3 times higher risk of pregnancy-associated with concomitant contraceptive implant and efavirenz- vs. nevirapine-containing ART use [7]. We also find that concomitant DMPA, the leading alternative contraceptive method, and efavirenz-containing ART use have a similar or higher risk of pregnancy than concomitant implants and efavirenz-containing ART use. Thus, in settings where efavirenz-containing ART remains a common ART option, ministries of health and programs should reconsider any restrictions on concomitant implant and efavirenz use and ensure that WLHIV have access to all available contraceptive methods. In settings where other ART options are available, such as dolutegravir-containing ART, women currently using or desiring implants and on efavirenz should be prioritized for a switch to dolutegravir-containing ART.

### Programmatic relevance of our findings

As dolutegravir-containing ART is increasingly used as first-line ART worldwide, including among women of reproductive potential, the hope is that dolutegravir will avoid any drug-drug interactions with hormonal contraceptives [31]. Early data from a pharmacokinetic study with dolutegravir and contraceptive implants supports this suggestion [32]. Nonetheless, for a small subset of WLHIV, regimens containing efavirenz or newer NNRTIs may still be their best option, due to enduring concerns with possible teratogenicity, intolerance to or weight gain with dolutegravir, drug-drug interactions with dolutegravir and other therapeutics, or increasing drug resistance to dolutegravir. Currently, a large number of women worldwide remain on efavirenz. Therefore, policymakers, program staff, and WLHIV need to remain vigilant about known or possible drug-drug interactions with NNRTIs, including efavirenz and hormonal contraception.

When reduced effectiveness of implants with concomitant efavirenz use was first reported, certain countries, such as South Africa and Malawi, moved swiftly to limit implant use among WLHIV on efavirenz [33]. As more balanced data emerged, that despite their reduced effectiveness, implants remained one of the most effective contraceptive methods for WLHIV using efavirenz, many countries reversed their course. Similarly, when a possible signal was associated with peri-conception dolutegravir use and neural tube defects in infants, many countries rushed to limit dolutegravir use among WLHIV of reproductive potential [6]. As more comprehensive data emerged, such as modeling studies showing that dolutegravir use would still lead to fewer maternal or infant deaths and mother-to-child HIV infections [34, 35], coupled with outcry from WLHIV and other advocacy organizations, some countries reversed course again to allow some WLHIV to continue dolutegravir use. There are three common lessons to be learned from both the efavirenz/implant and dolutegravir/neural tube defect issues: (1) to not react dramatically to early reports of potential negative implications, and to appreciate the implications more holistically, accounting for leading alternative options and downstream consequences; (2) to offer WLHIV counseling and options, using a person- or human rights-centered approach; and (3) to bring WLHIV into the decision-making process so that their voices and thoughts are adequately represented in ultimate policies.

### Importance of novel statistical techniques used

Our findings suggest that studies around pregnancy using routine clinical data can yield valid estimates and underscore the continued investments in health information systems, including in resource-limited settings. We used a 3-phase, largely random sampling design to repeatedly ascertain exposures of contraceptive method and ART regimen and incident pregnancy from data sources of EMR, chart review, and telephone interviews with WLHIV. Our work went beyond many data validation studies in that we used information learned in subsequent samples to adjust the initial point estimates, potentially improving the robustness of our findings. Additionally, we used state-of-the-art statistical methods, including IPW coupled with generalized raking, to account for our validation sampling strategy while gaining efficiency over traditional methods. The chart review results most closely approximated the EMR results, which is expected since the chart files are the source forms for the EMR data entry. However, our phone interview data yielded an overall higher pregnancy incidence, as well as for concomitant implant and efavirenz use relative to concomitant implant and nevirapine use. Possible explanations for this observation may include recall, sampling, or ascertainment biases, all leading to estimates away from the null. First, recall bias may exist as efavirenz is a newer antiretroviral compared to nevirapine, and residual confounding may persist despite our adjusting for calendar time. This would lead to differential misclassification away from the null, with higher pregnancy incidence reported among efavirenz vs. nevirapine and implant users. Second, because we relied on the EMR and chart files for telephone numbers, those on the newer antiretroviral of efavirenz may be more likely to have working numbers though numbers are updated routinely regardless of specific ART users. Contraceptive implants have been introduced more recently than DMPA too, so possibly secular trends may have led to differential sampling. Third, it is possible the research assistants differentially assessed pregnancy among women concomitantly using implants and efavirenz, as they referenced their chart notes to guide their phone interview. Nonetheless, while caution is advised in interpreting the telephone interview data as closest to the “truth,” the validation phases illustrate the overall robustness of the EMR data quality.

### Limitations

Despite this being the largest and most robust study to date on this topic, our study has additional potential limitations. First, we are assuming that data are missing at random, including for the subsampling; however, where data missing differentially by some unmeasured factor associated with both our exposure and outcome categories, this could bias our findings. Second, the phone interview sample was not randomly selected; we purposefully focused on certain combinations of exposure and outcome categories to most meaningfully inform our study objectives, as ascertaining exposure-pregnancy relationships by self-reports for some categories, e.g., for OCPs, still does not shed greater light on the failure of that method. Third, we asked women to recall information dating back as many as 6 years from the time of the interview, and potential recall bias may be avoided with prospective ascertainment. Fourth, as with all observational studies, residual confounding, unobserved confounding (e.g., our analyses were limited in the comorbidities or coinfections we could adjust for), and time-varying confounders (e.g., CD4 cell count, BMI, or WHO status that we did adjust for) that actually exist on the causal pathway between ART/contraceptive method and pregnancy may exist with our study. Different study designs and future work that develops methods to account for both time-varying confounding and validation datasets would be interesting. Lastly, our study does not ascertain adherence to either ART regimen or contraceptive method, though is an accurate reflection of real-world effectiveness data. Notwithstanding these limitations, the relative comparisons between exposure categories within each sample uphold earlier findings; the two validation phases underscore the overall robustness of EMR data quality; the generalizability of our findings to other resource-limited settings remains high; and both the 3-phase sampling and our statistical approaches add innovation for analyses conducted with EMR data.

## Conclusion

First, with more robust data quality, we confirm prior findings of reduced contraceptive effectiveness when contraceptive implants are concomitantly used with efavirenz-containing ART, and equivalent or higher effectiveness compared to leading alternative contraceptive methods such as DMPA. Second, our novel methodology using a 3-phase sampling data validation approach provides an innovative tool for other analyses to improve the robustness of EMR data quality. These findings provide policymakers, program staff, and WLHIV greater confidence in guiding their decision-making around ART and contraceptive options.

## Supplementary Information

**Additional file 1:.** Supplementary text, figures, and tables.

## Data Availability

Upon reasonable request and provision of appropriate documentation, such as study protocol, ethics approval, and signed data access agreement, the corresponding author will facilitate sharing of de-identified data, data dictionary, and other data elements. Analysis code is publicly available at https://github.com/gustavodecastro/ImplantEFV.

## Abbreviations

AIDS: Acquired immunodeficiency syndrome
aIRR: Adjusted incidence rate ratios
ART: Antiretroviral therapy
BMI: Body mass index
CDC: Center for Disease Control and Prevention
CTSA: Clinical and Translational Science Award
CI: Confidence interval
DMPA: Depomedroxyprogesterone acetate
EA-IeDEA: East Africa International Epidemiology Databases to Evaluate AIDS
EMR: Electronic medical record
FACES: Family AIDS Care & Education Services
HIV: Human immunodeficiency virus
IQR: Interquartile range
IUDs: Intrauterine devices
IPW: Inverse probability weighting
LEC: Less effective contraceptive
AMPATH: Moi Teaching & Referral Hospital/Moi University & Academic Model Providing Access to Healthcare
MEC: More effective contraceptive
NCI: National Cancer Institute
NCATS: National Center For Advancing Translational Sciences
NIAID: National Institute of Allergy and Infectious Diseases
NICHD: National Institute of Child Health & Human Development
NIMH: National Institute of Mental Health
NIDA: National Institute on Drug Abuse
NIH: National Institutes of Health
NNRTIs: Non-nucleoside reverse transcriptase inhibitors
NRTIs: Nucleos(t)ide reverse transcriptase inhibitors
OCPs: Oral contraceptive pills
PEPFAR: President’s Emergency Plan for AIDS Relief
PI: Protease inhibitor
TB: Tuberculosis
USAID: United States Agency for International Development
USA: United States of America
WLHIV: Women living with HIV
WHO: World Health Organization

## References

[1] UNAIDS (2018). Fact sheet – World AIDS Day 2018.

[2] Alkema L, Kantorova V, Menozzi C, Biddlecom A (2013). National, regional, and global rates and trends in contraceptive prevalence and unmet need for family planning between 1990 and 2015: a systematic and comprehensive analysis. Lancet..

[3] Emina JB, Chirwa T, Kandala NB (2014). Trend in the use of modern contraception in sub-Saharan Africa: does women’s education matter?. Contraception..

[4] Jacobstein R (2018). Liftoff: the blossoming of contraceptive implant use in Africa. Glob Health Sci Pract..

[5] Zash R, Makhema J, Shapiro RL (2018). Neural-tube defects with dolutegravir treatment from the time of conception. N Engl J Med..

[6] Mofenson LM, Pozniak AL, Wambui J, Raizes E, Ciaranello A, Clayden P (2019). Optimizing responses to drug safety signals in pregnancy: the example of dolutegravir and neural tube defects. J Int AIDS Soc..

[7] Patel RC, Onono M, Gandhi M, Blat C, Hagey J, Shade SB (2015). Pregnancy rates in HIV-positive women using contraceptives and efavirenz-based or nevirapine-based antiretroviral therapy in Kenya: a retrospective cohort study. Lancet HIV..

[8] Chappell CA, Lamorde M, Nakalema S, Chen BA, Mackline H, Riddler SA (2017). Efavirenz decreases etonogestrel exposure: a pharmacokinetic evaluation of implantable contraception with antiretroviral therapy. AIDS..

[9] Perry SH, Swamy P, Preidis GA, Mwanyumba A, Motsa N, Sarero HN (2014). Implementing the Jadelle implant for women living with HIV in a resource-limited setting: concerns for drug interactions leading to unintended pregnancies. AIDS..

[10] Pyra M, Heffron R, Mugo NR, Nanda K, Thomas KK, Celum C (2015). Effectiveness of hormonal contraception in HIV-infected women using antiretroviral therapy. AIDS..

[11] Scarsi KK, Darin KM, Nakalema S, Back DJ, Byakika-Kibwika P, Else LJ (2016). Unintended pregnancies observed with combined use of the levonorgestrel contraceptive implant and efavirenz-based antiretroviral therapy: a three-arm pharmacokinetic evaluation over 48 weeks. Clin Infect Dis..

[12] Vieira CSBM, de Souza RM, Brito MB, Rocha Prandini TR, Amaral E, Bahmondes L, Duarte G, Quintana SM, Scaranari C, Ferriani RA (2014). Effect of antiretroviral therapy including lopinavir/ritonavir or efavirenz on etonogestrel-releasing implant pharmacokinetics in HIV-positive women. J Acquir Immune Defic Syndr..

[13] Botsis T, Hartvigsen G, Chen F, Weng C (2010). Secondary use of EHR: data quality issues and informatics opportunities. Summit Transl Bioinform..

[14] Thiru K, Hassey A, Sullivan F (2003). Systematic review of scope and quality of electronic patient record data in primary care. BMJ..

[15] Duda SN, Shepherd BE, Gadd CS, Masys DR, McGowan CC (2012). Measuring the quality of observational study data in an international HIV research network. Plos One..

[16] Floyd JS, Heckbert SR, Weiss NS, Carrell DS, Psaty BM (2012). Use of administrative data to estimate the incidence of statin-related rhabdomyolysis. JAMA..

[17] Geng EH, Emenyonu N, Bwana MB, Glidden DV, Martin JN (2008). Sampling-based approach to determining outcomes of patients lost to follow-up in antiretroviral therapy scale-up programs in Africa. JAMA..

[18] Giganti MJ, Shaw PA, Chen G, Bebawy SS, Turner MM, Sterling TR (2020). Accounting for dependent errors in predictors and time-to-event outcomes using electronic health records, validation samples, and multiple imputation. Ann Appl Stat..

[19] Kiragga AN, Castelnuovo B, Schaefer P, Muwonge T, Easterbrook PJ (2011). Quality of data collection in a large HIV observational clinic database in sub-Saharan Africa: implications for clinical research and audit of care. J Int AIDS Soc..

[20] Kang’a SG, Muthee VM, Liku N, Too D, Puttkammer N (2016). People, Process and technology: strategies for assuring sustainable implementation of EMRs at public-sector health facilities in Kenya. AMIA Annu Symp Proc.

[21] Muthee V, Bochner AF, Kang’a S, Owiso G, Akhwale W, Wanyee S (2018). Site readiness assessment preceding the implementation of a HIV care and treatment electronic medical record system in Kenya. Int J Med Inform..

[22] Patel RC, Jakait B, Thomas K, Yiannoutsos C, Onono M, Bukusi EA (2019). Increasing body mass index or weight does not appear to influence the association between efavirenz-based antiretroviral therapy and implant effectiveness among HIV-positive women in western Kenya. Contraception..

[23] Ministry of Health, NASCOP. Guidelines for use of antiretroviral therapy in Kenya. Nairobi: NASCOP; 2011.

[24] Ministry of Health, NASCOP. Guidelines on use of antiretroviral drugs for treating and preventing HIV infection in Kenya 2016. Nairobi: NASCOP; 2016.

[25] White JE (1982). A two stage design for the study of the relationship between a rare exposure and a rare disease. Am J Epidemiol..

[26] Breslow NE, Chatterjee N (1999). Design and analysis of two-phase studies with binary outcome applied to Wilms tumour prognosis. J R Stat Soc C (Appl Stat)..

[27] Deville JC, Sarndal CE (1992). Calibration estimators in survey sampling. J Am Stat Assoc..

[28] Deville JC, Sarndal CE, Sautory O (1993). Generalized raking procedures in survey sampling. J Am Stat Assoc..

[29] Breslow NE, et al. Improved Horvitz–Thompson estimation of model parameters from two-phase stratified samples: applications in epidemiology. Stat Biosci. 1.1:32–49. 10.1007/s12561-009-9001-6 PMC2822363 20174455

[30] Seaman SR, White IR, Copas AJ, Li L (2012). Combining multiple imputation and inverse-probability weighting. Biometrics..

[31] Song IH, Borland J, Chen S, Wajima T, Peppercorn AF, Piscitelli SC (2015). Dolutegravir has no effect on the pharmacokinetics of oral contraceptives with norgestimate and ethinyl estradiol. Ann Pharmacother..

[32] Patel RC, Stalter R, Onono M, Brown E, Adeojo LW, Adhu CK, et al. Dolutegravir-containing ART does not reduce etonogestrel implant concentrations. CROI; March 8-11, 2020; Virtual (Boston)2020.

[33] Patel RC, Morroni C, Scarsi KK, Sripipatana T, Kiarie J, Cohen CR (2017). Concomitant contraceptive implant and efavirenz use in women living with HIV: perspectives on current evidence and policy implications for family planning and HIV treatment guidelines. J Int AIDS Soc..

[34] Dugdale CM, Ciaranello AL, Bekker LG, Stern ME, Myer L, Wood R (2019). Risks and benefits of dolutegravir- and efavirenz-based strategies for South African women with HIV of child-bearing potential: a modeling study. Ann Intern Med..

[35] Phillips AN, Bansi-Matharu L, Venter F, Havlir D, Pozniak A, Kuritzkes DR (2020). Updated assessment of risks and benefits of dolutegravir versus efavirenz in new antiretroviral treatment initiators in sub-Saharan Africa: modelling to inform treatment guidelines. Lancet HIV..

