# Supplementary material for: Pregnancies among women living with HIV using contraceptives and antiretroviral therapy in western Kenya: a retrospective, cohort study
Source: BMC Med. 2021 Aug 13;19:178. doi: 10.1186/s12916-021-02043-z (PMC8361857; doi:10.1186/s12916-021-02043-z)
Supplement: Supplementary file 1 — Additional file 1:. Supplementary text, figures, and tables. [file 12916_2021_2043_MOESM1_ESM.docx]

**Supplementary Text A: Methods**

*2.2.2. Outcome (continued)*

For 3,614 (29.3%) of 12,350 pregnancies at AMPATH and 903 (8.7%) of 10,401 pregnancies at FACES, the data needed to calculate the date of likely conception were unavailable. For these pregnancies, we used the median time from the date of likely conception to the initial detection of the pregnancy derived from the remainder of the cohort (5.3 and 4.3 months at AMPATH and FACES, respectively) to impute the date of likely conception.

For the overall cohort, there were 31,291 (12.3%) of 254,605 and 8,847 (9.4%) of 94,162 observations at AMPATH and FACES, respectively, that had a missing pregnancy status. We assumed these women were not pregnant during these periods.

*2.2.3. Covariates (continued)*

The number of living children, marital status, and education level were documented at enrollment in care, though marital status was time-varying at AMPATH. Age, CD4 cell count, WHO clinical stage, use of TB medications, and calendar time were also time-varying, with average age calculated for each observation period, CD4 cell count and WHO clinical stage documented closest to the start of each period, and use of TB medications documented at any point during the period. We used the documented body weight value closest to the start of the observation period and height during enrollment in care or closest to start of the observation period. BMI was calculated using weight in kilograms divided by height in meters^2^ at the start of each observation period, and considered time-varying. If weight was <30kg or height was <100cm, we replaced the value with a backward and then forward imputation, when such values were available. If the adjacent values were not available, we conducted multiple imputations to replace values of weight <30kg or height <100cm. See publications of related analyses for greater information on these covariates (1, 2).

*2.3. Data validation via three-phase sampling (continued)*

Details for each individual phase and its methods are summarized below.

*2.3.1. First-phase sample: electronic medical records*

The first-phase sample, or study cohort, consisted of eligible women contributing observation time to this analysis as recorded in the EMR. Routine clinical and demographic data were collected at the program-supported health facilities at enrollment and follow-up visits utilizing standardized, paper instruments, which trained data clerks transcribed into EMR systems supported by an OpenMRS platform. The first observation period for a woman began on the date of the woman’s first visit on or after January 1, 2011 (which may not necessarily be at the time of contraceptive method or ART initiation or at the time of enrollment in HIV care). An observation period ended when the woman changed her ART regimen category, stopped using a contraceptive method (or was noted to be using another contraceptive method), a combination of change in contraceptive method or ART regimen, was noted to be pregnant, reached her last clinical visit noted during the study period, or reached the end of the study period. Thus, each observation could span multiple clinical visits. If a visit was missing documentation of a contraceptive method or ART regimen, the prior documentation was carried forward. A woman with only one documented visit during our study period would not contribute data to this analysis. For periods not covered by clinical visits in our analysis, we assume the data are missing at random, which means that the chance that data are missing depends only on known characteristics of a study participant.

*2.3.2. Second-phase sample: manual chart review*

From the first-phase sample, we sampled a random subset of observations for the second phase for the manual chart review. *A priori* we decided to only conduct the second-phase sample for certain ART and contraceptive method categories. For ART categories, we only included observations in efavirenz-containing, nevirapine-containing, PI-containing ART regimens, or no ART; other ART categories were excluded due to few person-years in those categories. For contraceptive categories, we only included observations in implant, DMPA, MEC, or no contraceptive method categories. OCPs and LEC method categories were excluded as determining true exposure to these highly user-dependent methods is problematic and less meaningful for this validation study. After the second-phase sample was generated, we decided that the chart review would not be conducted at facilities where less than 100 observations were sampled or those that were inaccessible to the local study teams. This led to the exclusion of 15 AMPATH- and 12 FACES-supported facilities.

In order to generate the random subset stratified by program, we first categorized each observation period based on its combination exposure category of contraceptive method and ART regimen use and the primary outcome of whether a pregnancy occurred or not in that observation period. Therefore, we generated 32 total categories corresponding to combinations of contraceptive method, ART exposure and pregnancy status (or contraceptive-ART-pregnancy categories; **supplementary table 1**). We then chose the sampling fraction we wanted for each of these 32 categories. For example, because validating a pregnancy that occurred while using an implant, when the baseline failure rate of implants is so low, was central to the aim of this validation study, we chose to sample 100% of those observations which had a pregnancy noted while using an implant in the EMR, regardless of ART exposure. On the other hand, because observations when a woman was not using any contraceptive method and no pregnancy was noted were numerous, we chose to sample only 0.5% of those observations for study feasibility. Once an observation period was sampled for inclusion in the second-phase sample (“index” observation), we removed all other observation periods for that same woman from the first-phase dataset so that another observation period with her could not be sampled again for another category. Our second-phase sample consisted of approximately 7% and 12% of the women contributing data to the first phase from AMPATH and FACES, respectively.

Three research assistants (RAs), one with a clinical background and two with data backgrounds, at each program underwent training to extract and enter data from the manual chart review into a REDCap database. They first ascertained the primary exposures and outcomes on a paper monthly calendar form (**supplementary figure 2**) and later entered this information into our REDCap database. The RAs were blinded to the index observation or any other observations for that woman in the first phase, and, therefore, did not know what the primary exposures or outcomes were. When ascertaining data for any given woman, the RAs reviewed the medical chart for the entire study period (i.e. January 2011 to December 2015), regardless of what observation periods were included in the first phase data. We organized the chart review activity by generating lists of all charts intended to be reviewed by facility; every woman sampled for the chart review was assigned to the facility list based on her most recent visit noted in the EMR. At the start of the study, the study investigators conducted in-person supervision of the chart reviews to ensure all appropriate parts of a medical chart were being ascertained and interpreted correctly for data entry, and a second RA double checked data entry into REDCap against the calendar form for a select number of charts.

We successfully conducted a chart review for 2455 of 3643 (67.4%) and 2625 of 3757 (69.9%) of the index observations sampled for the second phase for AMPATH and FACES, respectively, for a total of 5080 charts reviewed. The leading reason for not conducting a chart review was inability to locate the paper chart for review. For this analysis, 4,971 reviewed charts are included; 109 were excluded as they did not pertain to the subset of exposure categories, such as combination ART regimens, analyzed in this analysis.

*2.3.3. Third-phase sample: telephone interviews*

From the chart reviews which were successfully completed for the second phase, we conducted telephone interviews with a nonrandom subset of the women. These women were selected for telephone interview based on the index observation in the first sample. The index observations where a woman was noted to be pregnant while using an implant, regardless of ART, were given the first priority in attempting phone calls. The second priority group was the index observations where a woman was noted not to be pregnant while using an implant, regardless of ART. The third and fourth priority groups were the index observations where the woman was noted to be using DMPA and pregnant or not pregnant, respectively. The study team generated periodic lists of priority second-phase sample participants to call for the telephone interviews as the chart reviews progressed based on these contraceptive-ART-pregnancy categories.

When the RAs called potential participants, they read a standardized telephone script to confirm the identity of the woman and obtain verbal consent to participate in the telephone interview. The RAs used the completed calendar forms from the chart review to guide their telephone interview, noting the participant responses on the respective section on the calendar form. They later entered these data into our REDCap database. The RAs attempted calling a potential participant a maximum of five times before deeming the participant unreachable for a telephone interview.

For the overall third-phase sample, we successfully conducted a telephone interview for a total of 1285 women, 494 of 2455 (20.1%) women at AMPATH and 791 of 2625 (30.1%) women at FACES, for the index observations which had a successful chart review completed. The leading reasons for not conducting a telephone interview were the telephone number being out of service or temporarily disconnected (56.7%), a working telephone number but no answer (16.1%), asked to call back later (13.1%), or a potential participant declining to participate in study (1.6%). A total of 1350, 598, 210, 70, and 21 telephone contacts were attempted and not successfully conducted for a first, second, third, fourth, and fifth times. A total of 398 women sampled for the third phase lacked any telephone numbers in the EMR or chart review, and, therefore, no attempts were made to reach these women by telephone. For this analysis, 1243 telephoned women were included; 42 were excluded as they did not pertain to the subset of exposure categories analyzed in this analysis.

*2.4. Statistical analysis (continued)*

Ignoring the nested structure of the sampling scheme and simply working with the three datasets independently may lead to biased estimates due to the over-sampling of women in certain contraceptive-ART-pregnancy categories. In addition, carrying out a validation study on a subset of the participants creates a missing data problem in which the validated variables are missing for those subjects not validated. To address both of these issues, we applied robust IPW methods to estimate aIRRs. For the IPW procedures, we first determined the probability of being selected for the manual chart review (or second-phase sample), denoted as *p_1_*, which was empirically estimated based on the observed proportions of women sampled in each of the 32 contraceptive-ART-pregnancy categories described above. Next, given that a woman’s chart was reviewed, we estimated the probability that she was selected for a telephone interview (or third-phase sample), denoted as *p_2_*. This was done using logistic regression models that included as covariates the categories used to define the priority telephone interviewing strategy (i.e., implant-ART-pregnant, implant-ART-not pregnant, DMPA-ART-pregnant, etc.). For data from women with complete validation (both chart validation and phone interviews) we then assigned a weight 1/*w*, where *w=p_1_p_2_* was the estimated probability of being fully validated in both sampling phases, and we then computed the aIRRs using a Poisson model. The idea behind this approach is to create virtual “copies” of the women with validated data, to represent the women without (missing) validation data. Under assumptions that missing validation data are missing at random (i.e., that selecting a woman for validation depends only on known characteristics) and that models for these probabilities are correctly specified, IPW estimates of the adjusted IRRs are consistent estimates for the full EMR dataset (first-phase sample). This means that the IPW estimates are virtually the same as those that would have been generated had all individuals in the sample been validated (3).

Although consistent, IPW estimators are often highly variable, resulting in wide confidence intervals (4, 5). We applied generalized raking techniques to improve the efficiency of the IPW estimates (6). Generalized raking uses auxiliary variables recorded on all women in the EMR (first-phase sample) to fine tune the inverse probability weights. Since auxiliary variables are fully observed in the EMR, we can calculate their totals and compare these totals to those obtained from IPW; the totals should be similar, because IPW estimators try to recover estimates in the original dataset, although they will almost never be exactly the same. Raking improves the precision of IPW estimates by perturbing the inverse probability weights as little as possible but constraining them such that the weighted totals of the auxiliary variables match their observed totals. Therefore, if auxiliary variables are correlated with the variables of interest, then these constrained weights will result in more precise (less variable) estimates. Others have shown that for a regression parameter, the influence function, which assesses the effect of removing an observation on the parameter of interest, is the optimal auxiliary variable (7). The influence function in our model, which is based on the validated contraceptive method, ART regimen, and pregnancy status, is unknown for those records that were not validated. However, the influence function using the unvalidated contraceptive method, ART regimen, and pregnancy status is highly correlated with the true influence function, and it is known for all women in the EMR. We therefore constrained the inverse probability weights with the estimated influence function derived from a Poisson model fit to the unvalidated data. Lastly, we then obtained raking estimates of the aIRRs by fitting a Poisson model to the fully validated data using these calibrated weights.

In the telephone interviews, women often indicated they were not on any ART when the chart review indicated they were on an ART regimen (35.2% of the time) or indicated they were taking ART medications but could not recall which type (3.7% of the time); for such observations with no ART or unknown ART type in the telephone interviews, we imputed the ART type from the chart review using the ART type closest to the start of the observation period. If the chart review indicated a change in exposure status due to a change in ART regimen, then that change was accounted for in the telephone interview as well, for example, by splitting the prior observation into two observations. We did not change the contraceptive method or pregnancy data in the telephone interview dataset, even if it conflicted with the chart review dataset.

**Supplementary Text B: Results**

*3.3 Overall pregnancy incidence, by each sampling phase*

In the EMR (first-phase) sample, 11,724 women had 12,896 incident pregnancies, including 1,172 repeat pregnancies, resulting in an aIR of 6.1 per 100 w-y (95% CI 5.9, 6.2). In the chart review (second-phase) sample, 1,355 women had 1467 incident pregnancies, including 112 repeat pregnancies, resulting in an aIR of 8.0 per 100 w-y (95% CI 7.5, 8.4). In the telephone interview (third-phase) sample, 634 women had 737 incident pregnancies, including 103 repeat pregnancies, resulting in an aIR of 11.8 per 100 w-y (95% CI 10.9, 12.7).

*3.4 Pregnancy incidence by contraceptive method, regardless of ART, by each sampling phase ignoring weighting by subsequent sampling phases*

Among implant users, the aIR was 2.2 (95% CI 2.0, 2.4), 3.0 (95% CI 2.6, 3.4), and 4.1 (95% CI 3.4, 5.1) per 100 w-y in the EMR, chart review, and telephone interview samples, respectively (**supplementary table 4**). Adjusted pregnancy incidence by implant type (e.g., etonogestrel, levonorgestrel, or unknown), were largely similar (**supplementary table 2**).

Among DMPA users, the aIR was 5.3 (95% CI 4.4, 6.3), 8.2 (95% CI 6.2, 10.8), and 12.4 (95% CI 8.0, 19.2) per 100 w-y in the first-, second-, and third-phase samples, respectively (**supplementary table 4**).

*3.5 Pregnancy incidence by ART regimen, regardless of contraceptive method, by each sampling phase ignoring weighting by subsequent sampling phases*

Among efavirenz users, the aIR was 6.1 (95% CI 5.9, 6.3) per 100 w-y, 7.7 (95% CI 6.4, 9.3) per 100 w-y, and 11.6 (95% CI 8.5, 15.9) per 100 w-y in the EMR, chart review, and telephone interview samples, respectively.

Among nevirapine users, the aIR was 6.1 (95% CI 5.9, 6.3) per 100 w-y, 8.0 (95% CI 7.4, 8.7) per 100 w-y, and 10.7 (95% CI 9.3, 12.3) per 100 w-y in the EMR, chart review, and telephone interview samples, respectively.

**Supplementary References:**

1. Patel RC, Onono M, Gandhi M, Blat C, Hagey J, Shade SB, et al. Pregnancy rates in HIV-positive women using contraceptives and efavirenz-based or nevirapine-based antiretroviral therapy in Kenya: a retrospective cohort study. Lancet HIV. 2015;2(11):e474-82.

2. Patel RC, Jakait B, Thomas K, Yiannoutsos C, Onono M, Bukusi EA, et al. Increasing body mass index or weight does not appear to influence the association between efavirenz-based antiretroviral therapy and implant effectiveness among HIV-positive women in western Kenya. Contraception. 2019;100(4):288-95.

3. Seaman SR, White IR, Copas AJ, Li L. Combining Multiple Imputation and Inverse-Probability Weighting. Biometrics. 2012;68(1):129-37.

4. Scott AJ, Wild CJ. Fitting regression models with response-biased samples. Can J Stat. 2011;39(3):519-36.

5. Lumley T. Connections between Survey Calibration Estimators and Semiparametric Models for Incomplete Data Discussion. Int Stat Rev. 2011;79(2):230-2.

6. Deville J-C, Särndal C-E. Calibration estimators in survey sampling. Journal of the American statistical Association. 1992;87(418):376-82.

7. Breslow NE, et al. Improved Horvitz–Thompson estimation of model parameters from two-phase stratified samples: applications in epidemiology. Statistics in Biosciences.1.1:32-49.

**Supplementary Figure 2: Monthly calendar form completed by research assistants when conducting the chart review and telephone interviews**

**
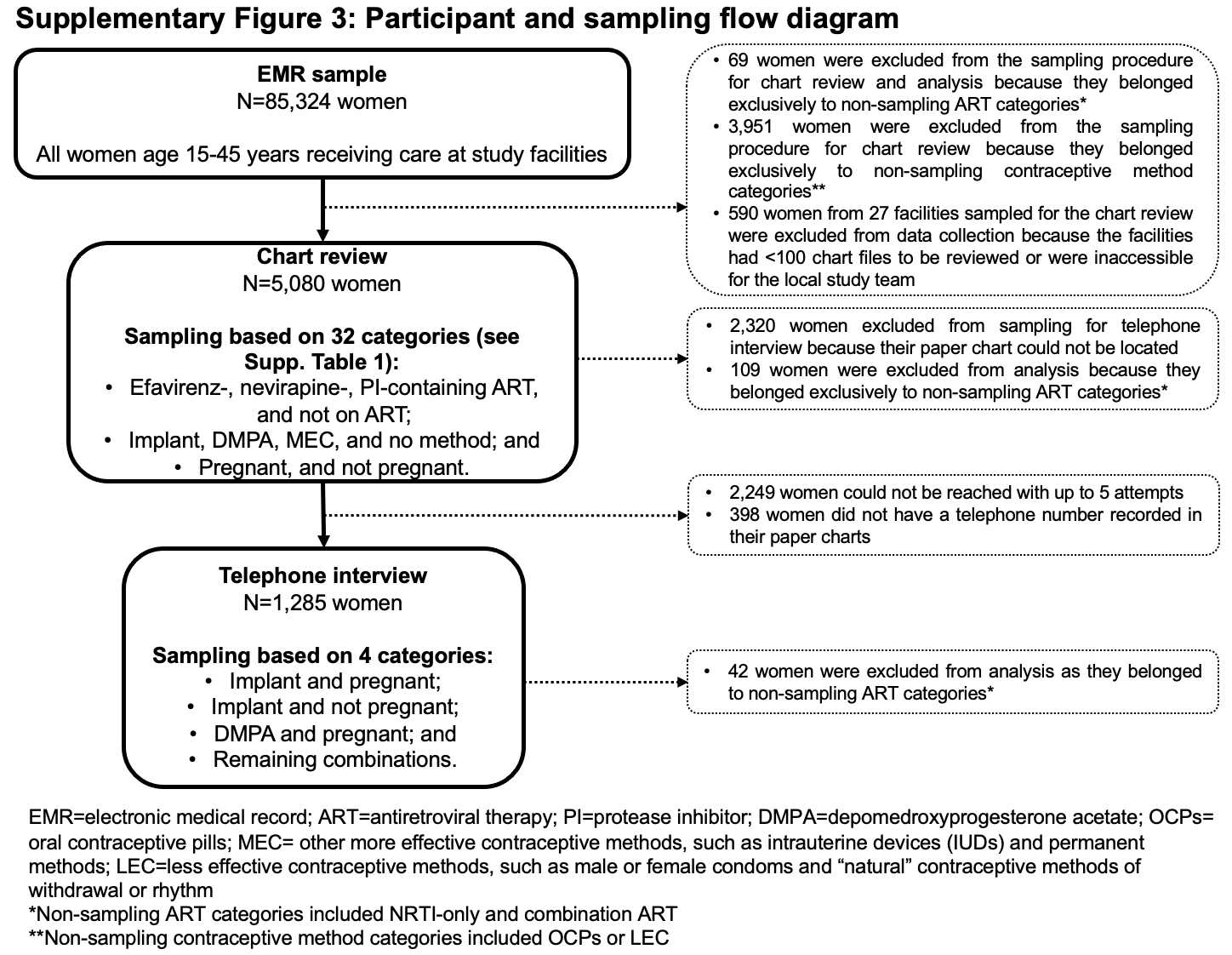
**

**Supplementary Table 1: Contraceptive method, ART regimen, and pregnancy status categories used for second (chart review) and third (telephone interview) phase sampling, stratified by program (AMPATH vs. FACES)**

| **Contraceptive method and ART regimen category** | **Number of observations with pregnancies (obtained from EMR data)** | | **Number of observations *without* pregnancies (obtained from EMR data)** | |
| --- | --- | --- | --- | --- |
|  | AMPATH | FACES | AMPATH | FACES |
| **Implant** | 144 | 322 | 13330 | 12656 |
| Efavirenz | 82 | 105 | 5300 | 3137 |
| Nevirapine | 35 | 114 | 5058 | 5537 |
| PI | 7 | 13 | 1014 | 751 |
| No ART | 20 | 90 | 1958 | 3231 |
| **DMPA** | 814 | 1333 | 30686 | 24239 |
| Efavirenz | 258 | 254 | 8830 | 5041 |
| Nevirapine | 298 | 642 | 12117 | 11006 |
| PI | 77 | 65 | 2390 | 1255 |
| No ART | 181 | 372 | 7349 | 6937 |
| **MEC** | 84 | 43 | 4142 | 4036 |
| Efavirenz | 25 | 8 | 1178 | 874 |
| Nevirapine | 34 | 19 | 1658 | 2013 |
| PI | 8 | 6 | 370 | 236 |
| No ART | 17 | 10 | 936 | 913 |
| **No method** | 3904 | 1253 | 127924 | 40876 |
| Efavirenz | 1174 | 244 | 38745 | 8812 |
| Nevirapine | 1579 | 549 | 50800 | 17288 |
| PI | 237 | 52 | 7699 | 1727 |
| No ART | 914 | 408 | 30680 | 13049 |

ART=antiretroviral therapy; EMR=electronic medical record; DMPA= depomedroxyprogesterone acetate; MEC= other more effective contraceptive methods, such as intrauterine devices (IUDs) and permanent methods; PI=protease inhibitor

**Supplementary Table 2: Crude and adjusted pregnancy incidence rate and rate ratios per 100 women-years (and 95% CI) by implant type and ART category, individually by each sampling phase ignoring validation sampling strategy (unweighted) and accounting for validation sampling strategy (weighted)**

| **Implant type & ART regimen category** | **Pregnancy** | **women-years** | **EMR** | | | **Chart review** | | | | **Telephone interview** | | |  |
| --- | --- | --- | --- | --- | --- | --- | --- | --- | --- | --- | --- | --- | --- |
|  |  |  | **aIR*** | **cIRR**** | **aIRR*** | **aIR*** | **cIRR**** | **aIRR* (unweighted)** | **aIRR*** (weighted)** | **aIR*** | **cIRR**** | **aIRR* (unweighted)** | **aIRR*** (weighted)** |
| **Etonogestrel implant** | 270 | 6035.8 | 2.5 (2.2,2.8) |  |  | 2.8 (2.1,3.6) |  |  |  | 4.0 (3.1,5.1) |  |  |  |
| **Nevirapine** | 91 | 2980.3 | 1.8 (1.5,2.3) | Ref | Ref | 1.9 (1.2,3.0) | Ref | Ref | Ref | 2.3 (1.3,3.8) | Ref | Ref | Ref |
| **Efavirenz** | 92 | 1352.9 | 3.9 (3.2,4.8) | **2.2 (1.6,2.9)** | **2.1 (1.6,2.8)** | 4.6 (3.2,6.6) | **2.6 (1.4,4.7)** | **2.4 (1.3,4.4)** | **3.2 (1.4,7.3)** | 7.6 (5.4,10.9) | **3.3 (1.8,6.3)** | **3.3 (1.8,6.3)** | **4.3 (1.2,15.6)** |
| **PI** | 13 | 361.4 | 2.2 (1.3,3.8) | 1.2 (0.7,2.1) | 1.2 (0.7,2.2) | 1.6 (0.4,6.5) | 0.9 (0.2,4.0) | 0.8 (0.2,3.7) | 0.6 (0.2,2.2) | 1.3 (0.2,9.2) | 0.5 (0.1,3.9) | 0.6 (0.1,4.4) | 1.4 (0.2,9.2) |
| **No ART** | 74 | 1329.8 | 2.4 (1.9,3.0) | **1.8 (1.3,2.4)** | **1.3 (1.0,1.8)** | 2.3 (1.1,4.8) | 1.5 (0.6,3.6) | 1.2 (0.5,2.9) | 1.1 (0.4,3.1) | 3.5 (2.1,5.6) | 1.7 (0.8,3.5) | 1.5 (0.8,3.1) | 3.3 (0.8,14.5) |
| **Levonorgestrel implant** | 40 | 1088.0 | 1.9 (1.3,2.8) |  |  | 3.0 (1.7,5.4) |  |  |  | 4.3 (2.4,7.8) |  |  |  |
| **Nevirapine** | 19 | 685.8 | 1.5 (1.0,2.3) | Ref | Ref | 2.7 (1.8,4.0) | Ref | Ref | Ref | 2.0 (0.9,4.7) | Ref | Ref | Ref |
| **Efavirenz** | 9 | 98.2 | 5.0 (2.8,8.9) | **3.2 (1.5,6.9)** | **3.3 (1.6,6.9)** | 4.6 (3.1,6.8) | **1.7 (1.0, 1.3)** | **1.7 (1.0,3.0)** | 1.9 (0.9,4.2) | 7.2 (4.4,11.7) | **3.9 (1.5,10.4)** | **3.6 (1.4,9.5)** | **3.1 (1.6,5.9)** |
| **PI** | 0 | 40.8 | -- | -- | -- | -- | -- | -- | -- | -- | -- | -- | -- |
| **No ART** | 12 | 263.2 | 1.9 (1.1,3.2) | 1.6 (0.8,3.3) | 1.3 (0.6,2.5) | 2.5 (1.3,5.0) | 1.1 (0.5,2.5) | 0.9 (0.4,2.1) | 0.7 (0.2,1.9) | 3.6 (1.6,8.2) | 2.0 (0.6,6.4) | 1.8 (0.6,5.9) | 1.5 (0.7,3.3) |
| **Unknown implant type** | 156 | 4849.3 | 1.8 (1.4,2.5) |  |  | 3.1 (1.8,5.4) |  |  |  | 11.5 (1.4,7.6) |  |  |  |
| **Nevirapine** | 86 | 1946.6 | 1.3 (1.0,1.8) | Ref | Ref | 1.8 (1.2,2.6) | Ref | Ref | Ref | 26 (3.1,215.6) | Ref | Ref | Ref |
| **Efavirenz** | 7 | 1793.4 | 2.5 (2.0,3.1) | **2.4 (1.6,3.4)** | **1.9 (1.3,2.7)** | 4.6 (3.6,5.9) | **2.9 (1.9,4.6)** | **2.6 (1.6,4.0)** | **2.4 (1.3,4.5)** | 0 (0,0) | 0 (0,0) | 0 (0,0) | 0 (0,0) |
| **PI** | 24 | 357.5 | 1.1 (0.5,2.4) | 0.9 (0.4,2.1) | 0.9 (0.4,1.9) | 3.1 (1.6,6.0) | 1.8 (0.8,3.9) | 1.7 (0.8,3.7) | 1 (0.3,3.4) | 0 (0,0) | 0 (0,0) | 0 (0,0) | 0 (0,0) |
| **No ART** | 39 | 751.8 | 1.6 (1.1,2.4) | 1.5 (0.9,2.5) | 1.2 (0.7,2.0) | 3 (1.9,4.7.0) | **1.9 (1.0,3.5)** | 1.7 (0.9,3.0) | 1.3 (0.6,2.6) | -- | -- | -- | -- |

ART=antiretroviral therapy; EMR=electronic medical record; aIR=adjusted incident rate; cIRR= “crude” incident rate ratio; aIRR=adjusted incident rate ratio; DMPA= depomedroxyprogesterone acetate; COC/OCPs= combined oral contraceptives (COCs) or oral contraceptive pills (OCPs); MEC= other more effective contraceptive methods, such as intrauterine devices (IUDs) and permanent methods; LEC=less effective contraceptive methods, such as male or female condoms and “natural” contraceptive methods of withdrawal or rhythm; PI=protease inhibitor

**Bolded** estimates indicate statistically significant findings.

*Calculated using Poisson models with interaction terms between ART and contraceptive method categories, program, various covariates (average age within the observation period, average age squared, marital status, education status, any number of living children, WHO clinical stage, CD4 cell count, square root of CD4 cell count, log BMI, square root of log BMI, use of any anti-tuberculosis medications, and calendar time), and robust standard errors. Unweighted analyses ignored the validation sampling strategy.

**Calculated using Poisson models, which included adjustment for an interaction term between ART and contraceptive method category and program.

***Calculated using IPW, generalized raking, and Poisson models with interaction terms between ART and contraceptive method categories, program, various covariates (average age within the observation period, average age squared, marital status, education status, any number of living children, WHO clinical stage, CD4 cell count, square root of CD4 cell count, log BMI, square root of log BMI, use of any anti-tuberculosis medications, and calendar time), and robust standard errors. Weighed analyses accounted for the validation sampling strategy.

**Supplementary Table 3: Adjusted pregnancy incident rate and rate ratios per 100 women-years (and 95% CI) by contraceptive method among efavirenz-containing ART users, individually by each sampling phase ignoring validation sampling strategy (unweighted) and accounting for validation sampling strategy (weighted)**

|  | | **EMR** | | **Chart review** | |  | **Telephone interview** | |  |
| --- | --- | --- | --- | --- | --- | --- | --- | --- | --- |
| **Efavirenz** | **aIR*** | | **aIRR*** | **aIR*** | **aIRR* (unweighted)** | **aIRR** (weighted)** | **aIR*** | **aIRR* (unweighted)** | **aIRR** (weighted)** |
| **Implant** | **3.1 (2.7,3.6**) | | Ref | **4.6 (3.8,5.5)** | Ref | Ref | **7.4 (5.6,9.9)** | Ref | Ref |
| **DMPA** | **5.6 (5.2,6.2)** | | **1.8 (1.5,2.1)** | **8.2 (6.8,9.9)** | **1.8 (1.4,2.3)** | 1.5 (0.9,2.4) | **12.3 (8.7,17.3)** | **1.7 (1.1,2.6)** | **2.4 (1.0,6.1)** |
| **ocps** | **10.9 (8.9,13.4)** | | **3.5 (2.7,4.5)** | **8.1 (3.8,17.0)** | 1.7 (0.8,3.8) | 1.3 (0.5,3.6) | 0 (0,0) | 0 (0,0) | - |
| **MEc** | **2.3 (1.6,3.2)** | | 0.7 (0.5,1.1) | 0.6 (0.1,4.3) | **0.1 (0.0,1.0)** | **0.0 (0.0,0.3**) | 0 (0,0) | 0 (0,0) | - |
| **LEc** | **8.0 (7.5,8.5)** | | **2.6 (2.2,3.0)** | **12.1 (9.3,15.6)** | **2.6 (1.9,3.6)** | 3.6 (1.9,6.7) | **23.9 (14.9,38.2)** | **3.3 (1.9,5.7)** | 1.9 (0.5,7.8) |
| **No method** | **5.8 (5.5,6.1)** | | **1.9 (1.6,2.2)** | **12.9 (10.0,15.1)** | **2.8 (2.2,3.6)** | 2.6 (1.5,4.5) | **16.7 (13.1,21.2)** | **2.3 (1.6,3.4)** | 2.1 (0.8,5.0) |

ART=antiretroviral therapy; EMR=electronic medical record; aIR=adjusted incident rate; aIRR=adjusted incident rate ratio; DMPA= depomedroxyprogesterone acetate; OCPs= oral contraceptive pills (OCPs); MEC= other more effective contraceptive methods, such as intrauterine devices (IUDs) and permanent methods; LEC=less effective contraceptive methods, such as male or female condoms and “natural” contraceptive methods of withdrawal or rhythm; PI=protease inhibitor

**Bolded** estimates indicate statistically significant findings.

*Calculated using Poisson models with interaction terms between ART and contraceptive method categories, program, various covariates (average age within the observation period, average age squared, marital status, education status, any number of living children, WHO clinical stage, CD4 cell count, square root of CD4 cell count, log BMI, square root of log BMI, use of any anti-tuberculosis medications, and calendar time), and robust standard errors. Unweighted analyses ignored the validation sampling strategy.

**Calculated using IPW, generalized raking, and Poisson models with interaction terms between ART and contraceptive method categories, program, various covariates (average age within the observation period, average age squared, marital status, education status, any number of living children, WHO clinical stage, CD4 cell count, square root of CD4 cell count, log BMI, square root of log BMI, use of any anti-tuberculosis medications, and calendar time), and robust standard errors. Weighed analyses accounted for the validation sampling strategy.

**Supplementary Table 4: Crude and adjusted pregnancy incident rate and rate ratios per 100 women-years (and 95% CI) by contraceptive method and ART category, individually by each sampling phase ignoring validation sampling strategy (unweighted) and accounting for validation sampling strategy (weighted)**

| **Contraceptive method & ART regimen category** | **Pregnancy** | **women-years** | **EMR** | | |  | **Chart review** |  | |  | **Telephone interview** | |  |  |
| --- | --- | --- | --- | --- | --- | --- | --- | --- | --- | --- | --- | --- | --- | --- |
|  |  |  | **aIR*** | **cIRR**** | **aIRR*** | **aIR*** | **cIRR**** | **aIRR* (unweighted)** | | **aIRR*** (weighted)** | **aIR*** | **cIRR**** | **aIRR* (unweighted)** | **aIRR*** (weighted)** |
| **Implant** | 466 | 11978.6 | 2.2 (2.0,2.4) |  |  | 3.0 (2.6,3.4) |  | |  |  | 4.1 (3.4,5.1) |  |  |  |
| **Nevirapine** | 149 | 5612.7 | 1.6 (1.4,1.9) | Ref | Ref | 2.1 (1.6,2.6) | Ref | Ref | | Ref | 2.3 (1.5,3.5) | Ref | Ref | Ref |
| **Efavirenz** | 187 | 3244.4 | 3.1 (2.7,3.6) | **2.2 (1.8,2.8)** | **1.9 (1.6,2.4)** | 4.6 (3.8,5.5) | **2.4 (1.8,3.3)** | **2.2 (1.7,3.0)** | | **2.3 (1.5,3.5)** | 7.4 (5.6,9.9) | **3.3 (2.0,5.6)** | **3.2 (1.9,5.4)** | **3.2 (1.8,5.7)** |
| **PI** | 20 | 759.7 | **1.6 (1.0,2.5)** | 1.0 (0.6,1.6) | 1.0 (0.6,1.6) | 1.9 (1,3.5.0) | 1.0 (0.5,1.9) | 0.9 (0.5,1.8) | | 0.6 (0.2,1.4) | 1.5 (0.4,5.9) | 1.7 (0.9,3.1) | 0.7 (0.2,2.8) | 0.9 (0.3,2.7) |
| **No ART** | 110 | 2344.9 | 2.1 (1.7,2.5) | **1.7 (1.4,2.2)** | **1.3 (1.0,1.6)** | 2.7 (1.9,3.8) | **1.6 (1.0,2.4)** | 1.3 (0.9,2.0) | | 0.9 (0.5,1.5) | 3.5 (2.3,5.3) | 0.6 (0.1,2.6) | 1.5 (0.8,2.8) | 1.9 (0.9,3.7) |
| **DMPA** | 2150 | 22749.3 | 5.3 (4.4,6.3) |  |  | 8.2 (6.2,10.8) |  |  | |  | 12.4 (8.0,19.2) |  |  |  |
| **Nevirapine** | 940 | 10827.1 | 5.3 (5.0,5.7) | Ref | Ref | 7.0 (6.0,8.2) | Ref | Ref | | Ref | 12.1 (9.2,16) | Ref | Ref | Ref |
| **Efavirenz** | 512 | 5000.9 | 5.6 (5.2,6.2) | **1.2 (1.1,1.3)** | 1.1 (0.9,1.2) | 8.2 (6.8,9.9) | **1.3 (1.0,1.6)** | 1.2 (0.9,1.5) | | 1.0 (0.6,1.8) | 12.3 (8.7,17.3) | 1.1 (0.7,1.6) | 1 (0.7,1.6) | 1.0 (0.3,2.9) |
| **PI** | 142 | 1468.7 | 6.0 (5.1,7.1) | **1.1 (1.0,1.4)** | 1.1 (0.9,1.3) | 10.6 (7.7,14.5) | **1.5 (1.1,2.2)** | **1.5 (1.1,2.1)** | | 1.3 (0.6,2.7) | 12.3 (7.3,20.8) | 1.3 (0.8,2.0) | 1 (0.6,1.8) | 0.8 (0.2,2.9) |
| **No ART** | 553 | 5403.0 | 4.6 (4.2,5.0) | **1.2 (1.1,1.3)** | **0.9 (0.8,1.0**) | 9.7 (8,11.7) | **1.8 (1.4,2.2)** | **1.4 (1.1,1.7)** | | 0.9 (0.5,1.4) | 12.1 (8.6,17.1) | 1.1 (0.6,2.0) | 1 (0.6,1.5) | 0.9 (0.3,2.4) |
| **OCPs** | 325 | 2252.7 | 8.8 (7.1,10.8) |  |  | 13.4 (9.1,19.8) |  |  | |  | 10.0 (4.6,21.9) |  |  |  |
| **Nevirapine** | 141 | 1061.4 | 8.8 (7.5,10.4) | Ref | Ref | 17.8 (12,26.6) | Ref | Ref | | Ref | 22.9 (9.8,53.7) | Ref | Ref | Ref |
| **Efavirenz** | 87 | 504.2 | 10.9 (8.8,13.5) | **1.3 (1.0,1.7)** | 1.2 (0.9,1.6) | 8.0 (3.8,16.9) | 0.5 (0.2,1.1) | **0.4 (0.2,1.0)** | | 0.2 (0.0,0.7) | 0 (0,0) | 0.7 (0.4,1.3) | 0 (0,0) | 0 (0,0) |
| **PI** | 13 | 135.7 | 5.9 (3.5,10.2) | 0.7 (0.4,1.3) | 0.7 (0.4,1.2) | 11.3 (4.4,28.8) | 0.6 (0.2,1.7) | 0.6 (0.2,1.8) | | 0.8 (0.2,2.6) | 16.7 (5.1,54.7) | 1.0 (0.6,1.7) | 0.7 (0.2,3.2) | 0.8 (0.2,3.1) |
| **No ART** | 83 | 547.5 | 7.4 (6,9.2) | 1.1 (0.9,1.5) | 0.8 (0.6,1.1) | 12.4 (7.4,20.9) | 0.8 (0.4,1.6) | 0.7 (0.4,1.3) | | 0.5 (0.1,1.4) | 5.9 (1.3,25.9) | 0.7 (0.3,1.8) | 0.3 (0.0,1.4) | 0.1 (0.0,0.7) |
| **MEC** | 127 | 6814.4 | 2.0 (1.5,2.6) |  |  | 2.7 (1.6,4.7) |  |  | |  | 2.9 (0.9,9.9) |  |  |  |
| **Nevirapine** | 53 | 3558.5 | 1.7 (1.3,2.3) | Ref | Ref | 2.7 (1.4,5.4) | Ref | Ref | | Ref | 2.1 (0.3,14.6) | Ref | Ref | Ref |
| **Efavirenz** | 33 | 1602.2 | 2.3 (1.6,3.2) | 1.4 (0.9,2.1) | 1.3 (0.9,2.0) | 0.6 (0.1,4.3) | 0.2 (0.0,1.8) | 0.2 (0.0,1.8) | | 0.3 (0.0,3.1) | 0 (0,0) | 0 (0,0) | 0 (0,0) | 0 (0,0) |
| **PI** | 14 | 498.7 | 2.8 (1.7,4.7) | **1.9 (1.1,3.4)** | 1.6 (0.9,2.9) | 7.6 (2.5,22.7) | 2.9 (0.8,10.1) | 2.8 (0.8,10.0) | | 1.9 (0.3,13) | 37.4 (5.6,251.6) | 2.3 (0.1,37.3) | 17.8 (1.2,266.6) | 24.7 (2.2,283.2) |
| **No ART** | 27 | 1134.1 | 1.9 (1.3,2.9) | **1.6 (1.0,2.5)** | 1.1 (0.7,1.8) | 3.5 (1.2,10.6) | 1.5 (0.4,5.5) | 1.3 (0.4,4.7) | | 1.4 (0.3,7.1) | 4.5 (0.7,30.8) | 12.1 (0.6,227.6) | 2.1 (0.1,32.5) | 5.7 (0.7,45) |
| **LEC** | 4482 | 41237.0 | 8.2 (6.8,9.9) |  |  | 14.5 (10.8,19.4) |  |  | |  | 29.7 (19.0,46.4) |  |  |  |
| **Nevirapine** | 2217 | 22263.2 | 8.4 (8,8.8) | Ref | Ref | 17 (14.5,20) | Ref | Ref | | Ref | 32.9 (24.9,43.4) | Ref | Ref | Ref |
| **Efavirenz** | 914 | 9020.6 | 8 (7.5,8.5) | 1.0 (0.9,1.1) | 1.0 (0.0.9,1) | 12.1 (9.3,15.7) | 0.8 (0.6,1.1) | **0.7 (0.5,1.0)** | | 1.5 (0.7,3.4) | 23.9 (14.9,38.2) | 1.1 (0.8,1.5) | 0.7 (0.4,1.3) | 0.7 (0.2,2.5) |
| **PI** | 260 | 2256.3 | 9.5 (8.4,10.8) | **1.4 (1.3,1.5)** | **1.1 (1**.0**,1.3)** | 6.7 (3.3,13.3) | 1.2 (0.8,1.6) | **0.4 (0.2,0.8)** | | 0.2 (0.1,0.6) | 26.8 (11.2,64.3) | 0.7 (0.4,1.3) | 0.8 (0.3,2.0) | 0.1 (0.0,0.9) |
| **No ART** | 1068 | 7583.2 | 7.6 (7.1,8.2) | **1.2 (1.0,1.3)** | **0.9 (0.8,1.0)** | 14.8 (11.2,19.6) | 0.4 (0.2,0.9) | 0.9 (0.6,1.2) | | 0.6 (0.2,1.5) | 27.3 (17.7,42.1) | **1.6 (1.3,2.1)** | 0.8 (0.5,1.4) | 0.3 (0.1,0.9) |
| **No method** | 5169 | 82939.8 | 6.1 (5.1,7.4) |  |  | 14.6 (11.0,19.4) |  |  | |  | 17.6 (11.6,26.8) |  |  |  |
| **Nevirapine** | 2128 | 40112.7 | 6.1 (5.9,6.4) | Ref | Ref | 16.9 (14.9,19.1) | Ref | Ref | | Ref | 16.2 (13.2,20) | Ref | Ref | Ref |
| **Efavirenz** | 1418 | 22797.9 | 5.8 (5.4,6.1) | **1.2 (1.1,1.3)** | **0.9 (0.9,1.0)** | 12.9 (11.0,15.1) | **0.8 (0.7,1.0)** | **0.8 (0.6,0.9)** | | 1.1 (0.7,1.8) | 16.7 (13.1,21.3) | 0 (0,0) | 1.0 (0.7,1.4) | 0.7 (0.3,1.8) |
| **PI** | 289 | 4924.9 | 6.2 (5.5,7.0) | **1.1 (1.0,1.3)** | 1.0 (0.9,1.1) | 10.8 (7.5,15.7) | 0.7 (0.5,1.1) | **0.6 (0.4,0.9)** | | 1.3 (0.6,2.8) | 10.6 (5.9,19.2) | 0.4 (0.1,2.4) | 0.7 (0.3,1.2) | 0.2 (0.0,0.9) |
| **No ART** | 1322 | 14915.6 | 6.5 (6.1,6.9) | **1.7 (1.5,1.8)** | **1.1 (1.0,1.1)** | 14.2 (11.6,17.3) | 1.0 (0.8,1.3) | 0.8 (0.7,1.1) | | 0.4 (0.2,0.7) | 18.8 (16.2,21.9) | 1.4 (0.3,6.2) | 1.2 (0.9,1.5) | 0.2 (0.1,0.6) |

ART=antiretroviral therapy; EMR=electronic medical record; aIR=adjusted incident rate; cIRR= “crude” incident rate ratio; aIRR=adjusted incident rate ratio; DMPA= depomedroxyprogesterone acetate; OCPs= oral contraceptive pills (OCPs); MEC= other more effective contraceptive methods, such as intrauterine devices (IUDs) and permanent methods; LEC=less effective contraceptive methods, such as male or female condoms and “natural” contraceptive methods of withdrawal or rhythm; PI=protease inhibitor

**Bolded** estimates indicate statistically significant findings.

*Calculated using Poisson models with interaction terms between ART and contraceptive method categories, program, various covariates (average age within the observation period, average age squared, marital status, education status, any number of living children, WHO clinical stage, CD4 cell count, square root of CD4 cell count, log BMI, square root of log BMI, use of any anti-tuberculosis medications, and calendar time), and robust standard errors. Unweighted analyses ignored the validation sampling strategy.

**Calculated using Poisson models, which included adjustment for an interaction term between ART and contraceptive method category and program.

***Calculated using IPW, generalized raking, and Poisson models with interaction terms between ART and contraceptive method categories, program, various covariates (average age within the observation period, average age squared, marital status, education status, any number of living children, WHO clinical stage, CD4 cell count, square root of CD4 cell count, log BMI, square root of log BMI, use of any anti-tuberculosis medications, and calendar time), and robust standard errors. Weighed analyses accounted for the validation sampling strategy.
